# Too much information: exploring technology-mediated abuse in higher education online learning and teaching spaces resulting from COVID-19 and emergency remote education

**DOI:** 10.1007/s10734-022-00947-0

**Published:** 2022-10-27

**Authors:** Helen Bovill

**Affiliations:** School of Education and Childhood, University of the West of England, Frenchay Campus, Coldharbour Lane, Bristol, BS16 1QY UK

**Keywords:** Blended, Hybridized, Synchronous and asynchronous, Cyber bullying, Cyber stalking, Emergency remote education, Digital civility and digital natives

## Abstract

During COVID-19, universities across the globe experienced a rapid requirement to move to online learning and teaching provision. This rapid move has been explored as emergency remote education (ERE). This paper reviews and presents some emerging literature regarding ERE, demonstrating how this created an environment where technology-mediated abuse could arise within the university context. Intentional and unintentional forms of technology-mediated abuse, within a global context, are considered with account of how intersectional characteristics can impact. The paper concludes with a set of provocations explored within an example framework. The provocations are given to situate ways of thinking which are facilitative of safer and more respectful use of technological spaces. Both the provocations and example framework aim to be useful critical tools for program and module teams to adapt in higher education institutions within the online sphere. The phenomenon of ERE is an opportunity to consider what can be learned with regard to management of technology-mediated abuse. However, a focus on ERE presents limitations in the paper because of the smaller number of academic sources at this time, due to recency of the COVID-19 pandemic.

## Introduction

This paper focusses upon rapid moves to online learning and teaching during COVID-19 and how this created an environment where technology-mediated abuse could arise within the international university context (Universities UK (UUK), 2020a, b). Universities UK (2020a, p.4) note “control, coercion, threats and stalking” between staff, students, and between staff and students had been exacerbated within universities by an “unanticipated jump online”, referring to abuse conducted through online platforms, as technology-mediated abuse. For example, the removal of previous safety nets such as face-to-face contact where abuse of this kind might be noted, and signposting to support given, was lessened due to remoteness. Different and unfamiliar modes of online communication operated at pace meant that rules around contact between students and between staff and students shifted. Therefore, the usual, known, and understood parameters of online communication, in this rapidly changed environment shifted too. Remote communication can put in place a form of disinhibition (Hladiʹkovaʹ & Hurajova, 2020) which may change the ways in which we might otherwise communicate with one another.

This paper explores intentional and unintentional technology-mediated abuse, to consider what universities can learn to help prevent this in online learning and teaching spaces. The aim of this paper is to consider how to create safer and more respectful online learning and teaching spaces, as universities emerge out of emergency remote education (ERE) mode (Bozkurt et al., 2020).

Online learning and teaching have many forms and definitions,

most of the terms (online learning, open learning, web-based learning, computer-mediated learning, blended learning, m-learning, (for ex.) have in common the ability to use a computer connected to a network, that offers the possibility to learn from anywhere, anytime, in any rhythm, with any means (Cojocariu, et al., 2014; in Dhawan, 2020, p. 6).

Online learning and teaching are sometimes referred to as blended learning which is debated in its definitions but has been defined by Graham (2006, p. 41) as learning which “combine face-to-face instruction with computer mediated instruction.” Hybridized learning and teaching are another term used and Driesen (2016) considers differences between blended and hybridized, noting that while blended may refer to the combination in some form of on and offline learning and teaching, hybrid refers to a wider mix, drawing from all possibilities of learning and teaching delivery which best suits the learner. In the rapid move to online learning and teaching, necessitated by social distancing during COVID-19, the terms synchronous and asynchronous have been widely adopted to refer to the learning and teaching that ensued within universities. Synchronous learning and teaching features live real time delivery of content where learners learn together and at the same moment in time. Synchronous learning occurs through a timetabled delivery via live mediums such as lectures, seminars, and workshops, delivered either online or offline. Asynchronous learning refers to a format where content is made available for learners to access remotely at various times and is not reliant upon live real time delivery (Dhawan, 2020).

Gurukkal (2021) notes the centrality of technology to learning and teaching since the disruption of COVID-19 and considers the multiple platforms which can “facilitate learning anytime, anywhere and from any source world-wide” (p. 7). This enables personalized education with flexibility and choice but it also breaks the link between students and institutional control and may present “critical barriers” (Gurukkal, 2021, p.12). One such critical barrier is the way in which a rapid move to online learning and teaching can intentionally or unintentionally facilitate technology-mediated abuse. Institutions, university staff, and students need a greater understanding of this context to manage this and to provide online space which is safer and more respectful.

A rapid move to online learning and teaching and its potential to facilitate technology-mediated abuse are concerns for all nations. COVID-19 necessitated a global turn toward online learning and teaching,

more than 1.5 billion enrolled students of all ages from all around the globe experienced interruption of education which equals nearly 90% of the global student population (UNESCO, 2020a, 2020b; UNICEF, 2020, in Bozkurt et al., 2020, p. 1).

The speed at which education was transferred online has been discussed in a global context as “emergency remote education” (ERE) (Bozkurt et al., 2020). Bozkurt et al. (2020) produced a report to respond to the COVID-19 pandemic which represents the reflections, lessons learned, and suggestions from higher education “of 31 countries across the world with a representation of 62.7% of the whole world population” (p. 1). ERE is described as different to remote education, prior to COVID-19, which Bozkurt et al. (2020) note as optional and able to be planned. Alternatively, ERE arising out of the COVID-19 pandemic was an obligation that had to be set in place at pace to survive a time of crisis. This has resulted in many issues of concern which are of a global nature. For example, Bozkurt et al. (2020) note that trauma, psychological issues, and anxiety have risen for staff and students. This has necessitated a need to develop communities of support to create safe spaces, and for staff to develop “soft” skills and competencies of a pastoral and supportive nature, enabling them to survive and support others through this time of crisis and beyond. Bozkurt et al. (2020) discuss how the pandemic demonstrated a need to develop a “pedagogy of care,”

over a need to teach the curriculum. Now, more than ever before, educators are thinking about learners beyond their role in the classroom to the difficulties they may be facing in their personal lives. This care and concern is an important trait that needs to be developed and strengthened as it is not only needed in times of crisis but always (p. 8).

This paper explores the impacts of ERE in terms of technology-mediated abuse in a global manner, where globally students have been out of universities and separated from peers and university staff. It will explore the implications of technology-mediated abuse for students, staff, and university institutions in learning and teaching online spaces. It will note who is impacted by this abuse and consider intersectional and multiple forms of abuse, alongside considering some platforms and contexts that abuse occurs within. Strategies for developing safer and more respectful technologically-mediated spaces, as institutions move beyond a post emergency response, will be a focus. This paper is guided by the following questions. During and after a time of “emergency remote education” (Bozkurt et al., 2020):

1. How has technology-mediated abuse manifested within higher education online learning and teaching spaces?
2. What are some of the contexts and platforms where university students and staff experienced technology-mediated abuse?
3. Who has been affected by technology-mediated abuse in universities, and how have intersectional characteristics impacted upon this?
4. What can universities learn from this to help ensure safer and more respectful use of technological spaces of learning and teaching for both students and staff?

## COVID-19 online learning and teaching and technology-mediated abuse

Online learning and teaching are now likely part of the new normal in universities across the globe (Ashour, et al., 2021; Maguire, et al., 2020; Pokhrel & Chhetri, 2021). Responding to the challenges of online learning environments will also need to be part of the new normal.

Technology-mediated abuse can refer to, for example, “cyber stalking, monitoring, online harassment, and humiliation” (Brown, et al., 2018; in Messing, et al., 2020, p.10). Cyber stalking uses electronic communication to stalk someone and is described as involving fixated and obsessive behavior and gathering information to monitor and discredit a victim (Paladin, 2015). This may be operated through technological platforms, such as Facebook, TikTok, WhatsApp, Twitter, email, text (for example); or online learning and teaching environments such as Blackboard Collaborate, Pebblepad, Udemy, Coursera, (for example); or widely used communication platforms such as Microsoft Teams.

Technology-mediated abuse can be operated through “cyber bullying” or “cyber mobbing” which has been characterized by using “information and communication technologies to encourage intentional, repetitive and hostile behaviour of an individual or a group aimed at harming others” (Belsey, in Hladiʹkovaʹ & Hurajova, 2020, p. 2). This includes,

behaviour that involves harassment, threat, humiliation, stalking or other negative behaviour of an individual or a group using the Internet, interactive and digital technologies or mobile phones (Hollaʹ, in Hladiʹkovaʹ & Hurajova, 2020, p. 2).

It is important to note the impact of technology-mediated abuse on learners’ academic performance. Prior to COVID-19 Al-Rahmi et al. (2019, p. 12) distributed a survey to 538 university students in Malaysia finding cyber stalking and cyber bullying “dampen the positive relationship between student academic performance and social media use for open learning.”

The purpose of intentional technology-mediated abuse is to cause harm and it is particularly pernicious when conducted by groups targeting individuals. Group targeting can include “internet pile-ons” (Universities UK, 2019, p.16) where groups gang up on one person through numerous messages. It can include “virtual mobbing” encouraging others to bully individuals through, for example, hashtags, or “doxing” which is sharing someone’s personal information online so that others can access it for harmful purposes (Universities UK, 2019, p.16). Online modes of learning and teaching may exacerbate and increase instances of technology-mediated abuse, in its various forms, because of both disinhibition and anonymity afforded in online spaces which do not have clearly defined social barriers. This lessening, or perceived lessening of social barriers, can result in a loss of restraint and impulse control and an increase in instinctive behaviors which is,

driven by an urge to pleasure or destructiveness (desire to enjoy something, desire to harm someone); exhibitionism, departure from reality and escape to the world of phantasy (Hladiʹkovaʹ & Hurajova, 2020, p. 3).

The previous definitions of technology-mediated abuse are predicated on intent to render harm, but technology-mediated abuse may also result from behavior which unintentionally leads to abuse where there is no intent to cause harm. This paper does not set out to measure different kinds of technology-mediated abuse. However, based on papers discussing a gap in training for educators during ERE (Ashour, et al., 2021; Bozkurt et al., 2020; Gurukkal, 2021; Pathak, 2021; Pokhrel, & Chhetri, 2021; Watermeyer, et al., 2020), it is reasonable to surmise that at least some technology-mediated abuse occurs from unintentional actions of institutions and educators who are ill-equipped for ERE. Equally, it may be the case that peers unintentionally operate technology-mediated abuse. Powell-Lunder (2019) clarifies that although online abuse is often conducted through malicious intent, it is possible for abuse to occur online without intent to cause harm. For example, a person posting online may think what they post is a joke that will be enjoyed by the target as well as others. However, the target may interpret it as harmful. Sathyanarayana et al., (2018, p. 1) note,

cyberbullying also can happen accidentally. The impersonal nature of text messages, instant messages, and e-mails makes it very hard to detect the sender's tone – one person's joke could be another's hurtful insult.

Seglias Pallas and Greenhall Furman (2021) consider other forms of inappropriate behavior which may be operated with or without intent in online learning and teaching spaces, such as spamming the chat with (unwanted, bulk) trash, messages, viruses, or repetitive words, direct messaging a member who does not want to chat privately, or sending unsolicited pictures in direct or indirect messaging. Inappropriate behavior may include, either privately or to the whole group, commenting on appearance of others, disparaging comments (for example, about gender or sexuality), or mis-gendering someone, or commenting on a person’s “race” or ethnicity, or political or religious beliefs. Inappropriate behavior in online learning and teaching spaces can also include sexual innuendos or sexist jokes, racist jokes, or publicly asking someone out on a date online, for example. It is important to note here too that some research finds that underrepresented students are more likely to be victims of harassment in online learning and teaching spaces. Prior to COVID-19, Gierdowski et al. (2020) conducted an annual study of undergraduate university students’ experiences of information technology and reported survey results form 16,162 respondents across 71 US university finding,

although most of the online harassment that all respondents told us they experienced occurs in environments used for personal, non-coursework purposes, more Black/African American (17%), Hispanic/Latinx (17%), and Asian/Pacific Islander (13%) students said they are harassed in environments or apps their institution provides or sponsors than white students (11%). Black students are also more likely than individuals of other races/ethnicities to encounter harassment on platforms that are recommended by their instructors (p. 4). 

At the beginning of the COVID-19 pandemic, Universities UK produced two briefings (2020a, b) responding to the increase in domestic violence and technology-mediated abuse within the UK context, noting,

the unanticipated ‘jump’ online may increase technology mediated violence and abuse between staff, students, or between staff and students. Communicating from private rather than public spaces (the home rather than the university), using technology such as private mobile phones, and an increased use of work based social media accounts to communicate to students may create a sense of social closeness that can be exploited (Universities UK, 2020a, p. 4).

Universities UK (2020a, p. 4) also noted issues around potential power dynamics which “may be less visible than in the classroom environment, and those being targeted may (wrongly) feel more to blame and feel less able to seek support.” The second Universities UK report (2020b) noted that online modes of learning and teaching also have the potential to result in removing previous safety nets found within university for those experiencing violence and abuse. Face-to-face opportunities for abuse to be recognized during the pandemic were lessened, removing this “lifeline” of support for survivors, such as signposting and access to services like counselling and reporting structures (Universities UK, 2020b). If more remote modes of learning continue for some students in a landscape of hybridized delivery, these safety nets remain compromised.

Universities UK (2020b) noted that though figures were not yet available, “media coverage during the pandemic indicates an increase in incidents of online lectures being interrupted with distressing comments, videos, or abusive, indecent images” (p. 7), for example, universities having to act because students posted explicit, disturbing, and violent pornography in online learning and teaching spaces (Batty, 2020, p. 1).

Institutions or lecturers have also, in some cases, insisted that students turn their cameras on during online learning and teaching, resulting in students sometimes feeling uncomfortable, or peers taking screen shots of other students or staff and then posting them on line in other forums such as Twitter (Bond & Phippen, 2021). Additionally, Bond and Phippen (2021) note that the use of cameras in personal space can potentially leave both students and staff vulnerable through accidentally sharing information they might want kept private. For example, photographs or identifiable outside landmarks which may enable identification of students or staff, leaving them locatable and therefore open to abuse. Students and staff may have various safeguarding reasons for not wishing to be seen on camera such as not wanting to be located by a former abuser or potential stalker. Bond and Phippen (2021, p. 3) note that in any policy insisting on a “camera on” approach, it is necessary to recognize “article 8 of the European Convention on Human Rights” as this “specifically refers to the right for respect for private and family life, home and correspondence.” Other statutory and non-statutory guidance should also be considered such as The General Data Protection Regulation (GDPR) for institutions within Europe. GDPR may also apply to institutions outside of Europe who have students that are from Europe using their online learning and teaching spaces. Within GDPR guidance, students signing in to online environments may need to have parts of this guidance conveyed to them, and platforms used need to be GDPR compliant. Additionally, GDPR means that necessary data only should be collected and personal, sensitive, and controlled data such as health status, religious, or political beliefs should not be stored and collected unless explicitly necessary. This restriction to data includes the collection of it in forms such as videos, screen shots, or chat boxes (The Digital Teacher, 2018). Other countries have different legislation and guidance which might also need to be considered when setting up and operating online learning and teaching spaces. For example, the USA has the Family Educational Rights and Privacy Act (FERPA) which can have consequences for any institution gaining private information on a student that can be gleaned through recorded material without proper consents in place (Sadler, 2020).

When policy from institutions and, or staff insists on “a camera on” approach to online learning and teaching sessions, this also has the potential to be harmful in terms of intersectionality. Insisting on “cameras on” demonstrates a lack of awareness of different socio-economic or ethnic contexts, and this can harm both educators and students. Literat (2021) conducted a study into the use of TikTok in relation to home learning and what can be viewed from cameras while learning, creating a data corpus from hashtag links on TikTok. Referring to a video series on the TikTok platform labelled “online classes in a Mexican household” Literat (2021) draws attention to videos depicting,

the interruptions from family members that students have to deal with while participating in online classes. With very few exceptions, these were overwhelmingly posted by minority students…reflecting structural inequalities and the intersectionality of ethnicity and class (p. 8).

Another video sourced by Literat (2021) on TikTok found comparisons between white and Hispanic students home learning environment depicting,

“white people taking online school” (showing him smiling peacefully at his computer, over a soundtrack of relaxing music) versus “Hispanics taking online school” (trying to silence his mother, who is yelling in Spanish, out of frame) (p. 8).

This is humorously put together on the TikTok platform, but it demonstrates inequity and inequality within online learning and teaching home learning environments. These and other videos depict disadvantage that would previously have been hidden from view and which can become a source of trauma, or a source for potential abuse, intentionally or unintentionally, by peers.

Literat’s (2021) study also found instances where staff home lives became a central focus in the online learning and teaching environment by students, and information misused. An example given is students sharing and discussing a white fridge in the background of an educator delivering online learning and teaching, and this being used to denote poverty. It became a site of discussion which included derogatory comments on the forum and was an invasion of that educator’s privacy (Literat, 2021, p. 9).

Literat also looked at a genre of videos posted by female TikTokers which,

depicted clean, bright study spaces in shades of white, pink, and rose gold, and prominently featured expensive brands such as Apple products and high-end makeup or clothing brands (Literat, 2021, p. 9-10).

This is an example which draws into sharp relief differences in socio-economic circumstances for students which can be a potential site of abuse and harm as students notice, and may comment upon, differences between them that might formerly have been hidden from view.

Literat (2021) represents a small corpus of emerging research in this area, as research on the issue of technology-mediated abuse in online learning and teaching spaces in university is not yet widely available. This is due to the recency of such changes occurring because of the COVID-19 rapid move to online learning and teaching. Therefore, for this paper to respond to this gap, it is necessary to refer to media reporting of technology-mediated abuse which highlights this as problematic and in need of urgent research. For example, Cheung (2021, p. 2) a Professor from a Hong Kong university has drawn attention to a form of cybermobbing,

“naming and shaming”, also known as doxing or “human flesh search” in China, in which the victim’s personal identity or contact details are released to the public, leaving them vulnerable to harassment, stalking and other abuse.

Doxing has also been identified by Universities UK (2019) and is developed here by Cheung who notes the serious consequences of this mob form of attack, because it tends to involve multiple offenses across multiple abusers which can be very hard for the victim to bear and also difficult to respond to. Cheung (2021) notes that online learning and teaching environments can be used to mitigate this kind of abuse through awareness raising and role playing and refers to this as “empathy training” where educators can ask students to consider the impact of doxing upon their own lives or the lives of those they care about.

Bozkurt and Sharma (2020) ask very relevant questions of universities as to the lessons to be learned from ERE during COVID-19 that institutions can bring to bear when they consider how to “keep learning in a safe learning ecology” (p. iii). They suggest it is paramount to focus not just on educational content but also on “teaching how to share collaborate and support” (p. iii) where empathy and care are a focus. In the larger scale report from Bozkurt et al. (2020) which assesses 31 countries and their education provision during COVID-19, they recognize the trauma, psychological pressure, and anxiety that learners and teachers experienced during ERE and consider the development of a “pedagogy of care, affection and empathy” (p. 4). This pedagogy recognizes the role that emotions play in the experience of online learning and teaching and prioritizes listening to students, particularly those experiencing disadvantage so that they do not become more disadvantaged,

A care approach to education pushes educators to recognize and address the diversity of students’ experiences and vulnerabilities, allowing them to be more receptive not only to the assumed needs of students but also their expressed and individual needs. This requires structures and practices that go beyond academia and prioritizes the emotional and psychological development and needs of students (Bozkurt et al., 2020, p. 4).

The next part of this paper will consider policy and guidance regarding online provision as universities move beyond ERE and crisis response. Provocations for online learning and teaching that facilitates safer and more respectful use of technological spaces will be made.

## Policy and guidance for online learning and risk management of technology-mediated abuse

Prior to COVID-19, Bond and Phippen (2019) from the University of Suffolk in the UK developed an online safeguarding self-review tool determining 4 levels of readiness from 0 to 3 on which universities could assess themselves rated; 0, reactive; level 1, basic; level 2, embedded; and level 3, holistic. In this tool, Bond and Phippen (2019) provide clear definitions for 23 features and levels related to online safeguarding, clustered into four groups of policy, education and training, technology, and practice. Also prior to COVID-19, Phippen and Bond (2020) conducted research into online harassment and hate crime in higher education institutions within the UK which consisted of two freedom of information (FOI) requests in June and September 2019 to 135 universities within the UK regarding institutional policy, incident recording, and training to deal with online harassment and hate crime. They received responses from 130 institutions and their main findings regarding online policy were,

this is not a sector that has established policy or practice to support students who might become victims of online abuse and harassment. We see pockets of good practice, but we also see the majority of institutions who have little by way of policy, practice, recording or training that is anywhere near an effective response. What focus there is lies on the protection of the institution, rather than the support of students (Phippen & Bond, 2020, p. 18).

Phippen and Bond (2020) noted low levels of recording incidents of abuse, and they state this means that universities are either not experiencing much abuse, that students are not reporting this, or institutions are not recording reports effectively. They state, from research conducted, it is most likely the latter. Phippen and Bond (2020, p. 18) note “a highly concerning lack of training around online abuse, with few universities providing specialist training.” Their research establishes an issue with both online abuse and responding to online abuse by UK universities even prior to ERE, as necessitated by COVID-19.

Since the COVID-19 pandemic, Bond and Phippen’s (2021) media article considers the impact on student welfare as a result of the rapid rush to online learning. In this article, they make specific recommendations for practice with regard to responding to technology-mediated abuse in online learning and teaching spaces. This includes, that any online recording should be explicitly consensual and in gaining this consent, it should be clear to students where online recordings will be posted, who will have access to the recordings and for how long. They also note that if students record online content, they must also be able to understand the universities safety and protocol around this and abide by it. Bond and Phippen (2021) also note that academic staff should not assume that students are “digital natives.” This is an area considered by Universities UK (2019, p. 20) who state,

young people are often assumed to be ‘digital natives’ (Cowie & Myers, 2017) because they operate freely in the online world, use technology routinely to carry out a wide range of everyday activities, and are likely to spend a significant proportion of their life online (Department for Education, 2019, pp. 5–6). However, the ability to recognise and respond appropriately to online harassment and other potential harms online should not be assumed.

Sladdin (2020) writing for the global International Law Firm in the technology sector, Pinsent-Masons, points to the misapprehension that students are “digital natives” and like (Universities UK, 2019) raises the argument that this view,

is misplaced, resulting in insufficient mechanisms being in place to tackle not only the risks of online harassment but also the protection of the wellbeing of students while accessing a provider's services (Sladdin, 2020, p. 2).

Universities UK (2019) note that, as young people spend increasing amounts of time in online space, harmful behaviors online are not always recognized as such as they may be viewed as a continuation of behaviors previously experienced in school and not addressed (Pörhölä, 2016). Additionally, as there is great inconsistency in the education that young people receive regarding online safety and behaviors prior to coming to university, it can be very difficult to know the level of understanding that students in university have of “digital civility and welfare” (Universities UK, 2019, p. 7). Digital civility is based upon the global Digital Civility Index which is developed by Microsoft to encourage online safety. It is based upon four digital civility challenge ideals and Microsoft produces Digital Civility Index Reports each year across 30 plus global geographies. These four digital civility ideals are,

- **Live the golden rule**
  I will act with empathy, compassion, and kindness in every interaction, and treat everyone with dignity and respect.
- **Respect differences**
  I will appreciate cultural differences and honor diverse perspectives. When I disagree, I will engage thoughtfully and avoid name calling and personal attacks.
**Pause before replying**
  I will pause and think before responding to things I disagree with. I will not post or send anything that could hurt someone else, damage someone’s reputation, or threaten anyone’s safety, including my own.
**Stand up for myself and others**
  I will tell someone if I feel unsafe, offer support to those who are targets of online abuse or cruelty, report activity that threatens anyone’s safety, and preserve evidence of inappropriate or unsafe behavior. (Microsoft, n.d. p. 1).

The gap in online knowledge for educators has been identified through the move to ERE, as a global issue for which training is seriously needed (Ashour, et al., 2021; Bozkurt et al., 2020; Gurukkal, 2021; Pathak, 2021; Pokhrel, & Chhetri, 2021; Watermeyer, et al., 2020). Phippen and Bond (2020) research into online harassment and hate crime in higher education institutions within the UK found severe shortfalls in knowledge and awareness regarding digital space for institutions too in policy, reporting, and training regarding online harassment and hate crime. Bozkurt et al. (2020) also found that globally, both higher education institutions and learning and teaching staff in higher education institutions have varying levels of knowledge regarding digital space.

In 2011 Bayne et al., wrote a manifesto for teaching online which was written as a short provocation at the time and has since been discussed more fully in its intent “to stimulate ideas about creative online teaching, and to reimagine some of the orthodoxies and unexamined truisms surrounding the field” (Ross, et al., 2019, p. 22). One of the statements of the manifesto was “can we stop talking about digital natives?” (Bayne et al., 2011, 2016). Ross and Bayne (2016) note that this statement can be read as a question or a plea but denotes the futility in essentialising either students or staff as such. The term digital native creates unhelpful and inaccurate assumptions around a “supposed lack of capacity for reflection and attention on the part of young people” and “the impossibility of anyone over a certain age truly belonging in digital spaces” (Ross & Bayne, 2016, p. 1–2).

Additionally, the manifesto sought to consider time and space through another of its short statements, “distance is temporal, affective, political: not simply spatial” noting that because space, or where online learning and teaching take place, is often the priority; other aspects are given less attention (Ross & Bayne, 2016, p. 2). This paper finds similarly noting that “distance” in online learning and teaching is much more than spatial. Distance also poses issues around emotional distance to consider, such as how students and staff feel about using and delivering online learning and teaching, and how they feel about being able to cope with supporting those who are subject to extra stress and mental health issues, which technology-mediated abuse can contribute to. Wray and Kinman (2021) conducted a survey examining working life in UK Higher Education Institutions which included consideration of the challenges that COVID-19 had brought about. They received two thousand and forty-six academic and academic-related staff responses regarding “the psychosocial hazards they encounter, how they feel about the tasks they do and the availability and usefulness of support mechanisms to manage their wellbeing” (Wray & Kinman, 2021, p. 3). This report identified that during COVID-19, numbers of students experiencing stress and mental health issues had increased, along with the need to provide pastoral support. Some of the report findings demonstrate that staff do not necessarily feel resourced or equipped to offer the high levels of emotional support required or do not feel that they have the training and support to fulfil this aspect. Some feedback in the report noted increased demand from students which caused extra stress for staff responding to this, “students demand much more from you than they ever did: e.g. one emailed me 11 times one day as they had nobody else to talk to”; and “Students are experiencing more difficulties so need far more support both academically and emotionally” (p. 38). The report also noted the extra time this demands of staff as you cannot just chat at the end of a class; instead, online meetings have to be specifically arranged. Amongst recommendations from the report were a need for “adequate training and support for technology use” (p. 5). Bozkurt and Sharma (2020, p. iii) note that,

in a time of crisis, when people are under trauma, stress and psychological pressure, should we focus on teaching educational content or should we focus on teaching how to share, collaborate and support? We should remember, when things go back to normal, people will not remember the educational content delivered, but they will remember how they felt, how we cared for them, and how we supported them.

Distance impacts upon many facets of relations and relationships in online learning and teaching spaces for students, staff, and institutions. This is a complex and nuanced area to consider which requires critical approaches and debate amongst all of the communities involved. This is important during ERE but also in the aftermath as times of crisis may re-occur at any time given the ever-changing world we inhabit (Bozkurt & Sharma, 2020).

## Provocations for critically thinking about safer more respectful use of technological spaces

As a result of ERE and COVID-19, the UK Government produced “Guidance for safeguarding and remote education during COVID-19” (Gov.UK, 2020) . This was aimed at schools and colleges of further education and not specifically at higher education institutions but nevertheless is a starting point for guidance to plan and deliver online learning and teaching which aims toward safer more respectful use of technological spaces. Gov.UK (2020) included guidance for schools and colleges to review existing safeguarding and online policies so that they reflected online learning. Communication with students, to emphasize the importance of online safety, was recommended, as was encouraging students to speak up about anything worrying on line. Schools and colleges were advised to have and to communicate clear reporting routes. It was also stated by the UK Government that online content did not have to be recorded and that schools and colleges were best placed to make decisions regarding the policy they adopted in relation to live lessons and recording.

Universities UK (2020b) also noted of universities that with the increasing use of technology to deliver learning and teaching content online, there is a “need to support students and staff to engage with technology safely and to raise awareness of the potential harms from online harassment” (p. 10). Universities UK (2020b) stated that universities essentially needed “IT usage policies and clear information on expected behaviors in the online sphere” (p. 19).

Sladdin (2020) note the lack of statutory policy to guide higher education providers to outline their duties to be responsible for student safeguarding, given students’ status as adults. However, Sladdin (2020) states that higher education providers have a contractual duty of care to safeguard their students. In reality, the legal strength of that duty has not been properly tested but, higher education institutions need to be aware that in providing further services such as digital platforms, if they fail to monitor the impact of these upon student mental health, they can be in breach of this duty of care,

in cases where a student becomes the subject of abuse, or is at risk of abuse, within an online environment provided by their higher education provider, the institution needs to be able to demonstrate due diligence. This might involve, for example, using well-defined 'acceptable usage' policies, and implementing appropriate monitoring approaches and effective staff training to recognise and support those students at risk (Sladdin, 2020, p. 3).

The briefing from Pinsent Masons, written by Sladdin (2020), advises that higher education institutions set clear online conduct guidelines for students and staff in delivering online learning and teaching. This guidance should refer to online harassment in disciplinary policies and procedures and to the institutions’ student code of conduct. Sladdin’s (2020) briefing also states that students and staff must clearly understand the boundaries of acceptable behavior online and what the consequences are if those boundaries are overstepped by any parties.

Bayne et al., (2011, 2016) developed their Manifesto for teaching online as a generative tool for teachers in higher education to use to consider and critique their online practices and processes. The provocations set out next are offered in the same way: to be useful, generative, and to support critical engagement. They are not intended to replace a more comprehensive review, such as Bond and Phippen’s (2019) institutional audit tool. Alongside these ten provocations, the following statements may be useful to guide thinking about how to shape safer and more respectful spaces for online learning and teaching in potentially new landscape of higher education beyond ERE:

## Statements

- What does it feel like to use the online learning and teaching spaces for students, staff, and institutions?
- Which vulnerabilities are exposed in the online learning and teaching spaces for students, staff, and institutions?
- How do intersectional characteristics such as class, gender, ethnicity, culture, sexuality impact in the online learning and teaching spaces for students, staff, and institutions?

## Ten provocations

1. What specific policies of safer and more respectful online usage and delivery exist already in the institution and how do they relate to practice?
2. Do report and support structures exist in the institution and can they mediate online abuse?
3. What is the role of induction for students and staff in online usage and delivery?
4. What is the place of consultation in online policy and practice design?
5. How does compliance and data protection influence online decisions?
6. What is the online impact of cameras, chat boxes, comments, and other forms of real time communication?
7. How do students and staff understand consent online?
8. What online training needs do students and staff have?
9. How is online safeguarding prioritized and by whom?
10. How do students and staff champion and role model safe and respectful online usage and delivery?

A potential example of how to begin to operationalize this into a framework for program and module teams is given in Table 1 below.

**Table 1:** An example framework for program and module teams to inform online usage and delivery planning at the local level with regard to safer and more respectful use of technological spaces

Online usage and delivery framework questions	Potential responses
What does institutional and local online usage and delivery policy set out?	Is there specific university policy already in existence? What does it say that this team can use in their planning? How can this be communicated to the team? What gaps are there? How relevant is this policy? What is statutory?
What induction for online usage and delivery is there for new students/staff?	What is included in induction? How can we find this out? What is not currently included and how can we underpin this in the interim?
What training is there for online usage and delivery for students/staff?	What are the training needs in this specific area? What existing training can support this? How can we find out about unknown training needs and how can we respond to these?
What compliance and data protection needs to be considered at the local, national and international level?	How do we find out about our institutional responsibilities? Which parts of this compliance stand out most strongly for our particular program/module? How can we convey this to students/staff? What is statutory? What is not covered? Who might be exposed through our practices?
What is the impact of online communication in real time and after?	How do we best use cameras in the online space? What do students/staff need to be aware of in the use of comment boxes, chat spaces etc.? How will students/staff communicate to each other when using online spaces? Do all those using the space understand where any recordings sit and the parameters to their usage? Can any of our communication modes have unintended harmful consequences? How can we mitigate for these?
What safeguarding measures are needed in the online usage and delivery?	What safeguarding does the university already have? What gaps are there and how can we respond? Are there report and support structures in place and do students/staff know about them and how to use them? How can we convey this information in an accessible way? Are vulnerabilities, inequalities, inequities likely to be exposed throughout our practices? Do students/staff understand disclosure and support processes?
Is consent online understood by students/staff?	What university policy exists to support this and how can we use it? What consent messages are essential to convey to students/staff and how can we do this? Do students/staff understand consent and unintended or intended infringements upon this that can arise online?

In setting up a framework for safer and more respectful use of technological spaces, it is also useful to consider if there are pockets of practice that exemplify this and that can be role modelled or championed. As noted by Cheung (2021), awareness raising and role playing which they refer to as “empathy training” can be utilized by involving students and staff who have more knowledge and experience in these areas. This also aligns with Bozkurt et al. (2020) who consider an urgent need for developing a “pedagogy of care,” consisting of practices which extend beyond curriculum to the pastoral care and support of students and staff.

## Conclusion

Technology-mediated abuse and its issues are not equally felt across the globe. As Bozkurt et al. (2020) note, there are issues of equity within countries and systems and between countries and systems in terms of the response to ERE. There are lessons to be learned regarding the skills and competencies needed for crisis such as COVID-19 and rapid online learning and teaching, which will be felt differently by global nations. Some of which will not seek “to return to normal but to use this crisis as an opportunity to fix an education system that was already broken to begin with” (Black, 2020, in Bozkurt et al., 2020, p. 6).

In reality, higher education providers across the globe have struggled to respond to ERE (Bozkurt et al., 2020). The academic papers now emerging from this rapid obligation to move learning and teaching online have pointed toward a lack of training for staff generally in providing online learning and teaching (Ashour, et al., 2021; Bozkurt et al., 2020; Gurukkal, 2021; Pathak, 2021; Pokhrel, & Chhetri, 2021; Watermeyer et al., 2020). In addition to this, higher education providers were struggling prior to COVID-19 to provide adequate policies and guidance for safer and more respectful online spaces (Phippen & Bond, 2020).

There is also a great deal of misinformation regarding the digital awareness of safer and more respectful space by young people who are considered to be “digital natives” when in fact “digital civility” varies enormously (Bayne, et al., 2011, 2016; Microsoft, n.d.; Ross & Bayne, 2016; Ross, et al., 2019; Sladdin, 2020; Universities UK, 2019). Black (2020) offers up a different opinion of what COVID-19 could bring the education system globally, suggesting ERE could offer a breathing space to look back on how things are to how things could be. Through longer-term planning and review of lessons learned from ERE, safer and more respectful online space in international higher education institutions may result.
